# Incidence, determinants and perinatal outcomes of near miss maternal morbidity in Ile-Ife Nigeria: a prospective case control study

**DOI:** 10.1186/1471-2393-13-93

**Published:** 2013-04-15

**Authors:** Ikeola A Adeoye, Adedeji A Onayade, Adesegun O Fatusi

**Affiliations:** 1 Department of Epidemiology and Medical Statistics, College of Medicine, University of Ibadan, Ibadan, Nigeria; 2 Department of Community Health, College of Health Sciences, Obafemi Awolowo University, Ile-Ife, Nigeria

**Keywords:** Near miss maternal morbidity, Maternal health, Pregnancy complications, Perinatal outcomes, Nigeria

## Abstract

**Background:**

Maternal mortality ratio in Nigeria is one of the highest in the world. Near misses occur in larger numbers than maternal deaths hence they allow for a more comprehensive analysis of risk factors and determinants as well as outcomes of life-threatening complications in pregnancy. The study determined the incidence, characteristics, determinants and perinatal outcomes of near misses in a tertiary hospital in South-west Nigeria.

**Methods:**

A prospective case control study was conducted at the maternity units of the Obafemi Awolowo University Teaching Hospitals Complex, Ile-Ife Nigeria between July 2006 and July 2007. Near miss cases were defined based on validated disease-specific criteria which included severe haemorrhage, hypertensive disorders in pregnancy, prolonged obstructed labour, infection and severe anemia. Four unmatched controls of pregnant women were selected for every near miss case. Three categories of risk factors (background, proximate, clinical) which derived from a conceptual framework were examined. The perinatal outcomes were also assessed. Bi-variate logistic regressions were used for multivariate analysis of determinants and perinatal outcomes of near miss.

**Results:**

The incidence of near miss was 12%. Severe haemorrhage (41.3%), hypertensive disorders in pregnancy (37.3%), prolonged obstructed labour (23%), septicaemia (18.6%) and severe anaemia (14.6%) were the direct causes of near miss. The significant risk factors with their odds ratio and 95% confidence intervals were: chronic hypertension [OR=6.85; 95% CI: (1.96 – 23.93)] having experienced a phase one delay [OR=2.07; 95% CI (1.03 – 4.17)], Emergency caesarian section [OR=3.72; 95% CI: (0.93 – 14.9)], assisted vaginal delivery [OR=2.55; 95% CI: (1.34 – 4.83)]. The protective factors included antenatal care attendance at tertiary facility [OR=0.19; 95% CI: (0.09 – 0.37)], knowledge of pregnancy complications [OR=0.47; 95% CI (0.24 – 0.94)]. Stillbirth [OR=5.4; 95% CI (2.17 – 13.4)] was the most significant adverse perinatal outcomes associated with near miss event.

**Conclusions:**

The analysis of near misses has evolved as a useful tool in the investigation of maternal health especially in life-threatening situations. The significant risk factors identified in this study are amenable to appropriate public health and medical interventions. Adverse perinatal outcomes are clearly attributable to near miss events. Therefore the findings should contribute to Nigeria’s effort to achieving MDG 4 and 5.

## Background

Worldwide, more than half a million women between the ages of 15 and 49 years die each year from the complications of pregnancy and childbirth [1,2]. Developing countries disproportionately bear this burden inspite of intensive global attention and efforts [3]. Near misses have emerged as a useful complement to the investigation of maternal deaths [4-6]. A near miss is defined as a woman who nearly died but survived a complication that occurred during pregnancy, childbirth or within 42 days or termination of pregnancy [7-9]. The study of near misses, which occur in far greater numbers than maternal deaths, allows for a more robust quantification and conclusion on the risk factors and determinants of life – threatening complications [4,7]. Several studies have suggested that identification of risk factors of severe morbidity may contribute to maternal mortality reduction by ascertaining those factors that are modifiable by appropriate medical and public health interventions [10-13].

The predictors of maternal morbidity have been categorized into three groups [14,15]: those not amenable to change such as race; those that might be amenable to social change for instance barriers in the utilization of health services and clinical factors which respond to medical interventions. The quality of medical care and socio-environmental factors are important determinants of maternal outcomes in life threatening situations. In the United Kingdom [10] for example, the main predictors of near misses were: age over 34 years, non- white ethnic group, past or current hypertension, previous postpartum haemorrhage, delivery by emergency caesarian section, antenatal admission to hospital, multiple pregnancy, social exclusion and iron or anti-depressants use at antenatal booking. In fact, wide disparity in maternal morbidity and mortality levels between developed and developing countries may be attributable to some of these factors.

Usually the health of mothers and their newborn are inseparable. Perinatal outcomes refer to life events that occur to a newborn infant between the age of viability (i.e. after 28 weeks of gestation) and the first week of life. Studies have found that maternal complications have higher risk of adverse perinatal outcomes like stillbirth, birth asphyxia and neonatal deaths [16-18]. Generally, a significant proportion of the deaths that occur in under-five children (estimated as 7.6 million in 2010) take place in the first month of life with about two thirds occurring in the first week and the highest risk on the first day of life [19]. And just like maternal mortality, 98% of these deaths is unduly borne by developing countries especially sub-Saharan Africa with the highest risk of neonatal deaths globally. The main direct causes of perinatal death are preterm delivery (28%), Sepsis (26%), birth asphyxia (23%) and others.

**Figure 1:**
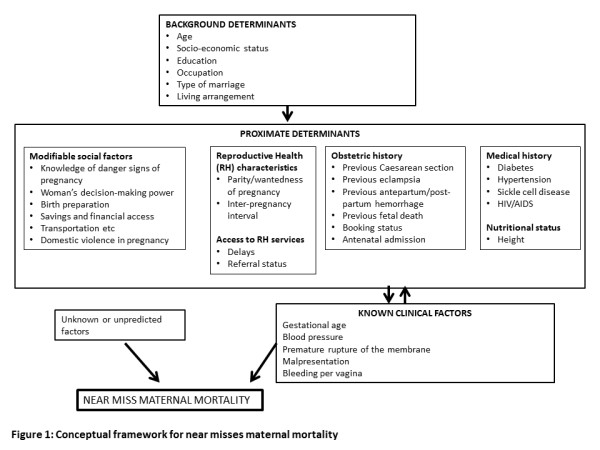
Conceptual framework for near misses maternal morbidity.

Studies on near misses have been scarce in Nigeria, despite her high maternal death burden. With a maternal mortality ratio of 840 per 1000000 live births [20], Nigeria has one of the highest maternal mortality ratio and with a large population of over 160 million, Nigeria records an estimated 40,000 maternal deaths annually – the second highest in the world. The child health indices even though has been on the decline since 1990, are also disproportionately higher in Nigeria compared to several other low income countries: the neonatal mortality and under-5 mortality rates are 91 per 1000 live-births and 145 per 1000 live-births respectively [19]. Therefore, studies on maternal mortality related events like near miss and perinatal outcomes in Nigeria is crucial to further understanding associated issues and to provide evidence based platform for appropriate interventions. Only one study was found to have been published regarding near miss in Nigeria when we conducted a search using PUBMED [21]. The study, however, only focused on pattern of near miss without considering the determinants. In addition, like many other investigation of near miss, [21-24] the Nigerian study used a retrospective approach, which may have the challenges of bias, lack of information on important confounding variables and incomplete information from poor documentation. In contrast, the current study is a prospective investigation of near misses occurring in a tertiary hospital in south western Nigeria. It documents the incidence and characteristics of near misses over a one year period using a three-level conceptual framework (Figure 1); the framework was based on the work of Reynold and collegues [25] who investigated near miss maternal events in Senegal and is an adaptation of the framework originally developed by McCathy and Maine. The framework facilitated the identification of critical associated factors at the level of patient, socio-environmental and health systems. Our study also examined the perinatal outcomes associated with life-threatening maternal morbidity in Nigeria.

## Methods

### Study setting

The study, a prospective case control study, was carried out at the Obafemi Awolowo University Teaching Hospitals Complex (OAUTHC), Ile-Ife, South-Western Nigeria from July 2006 to June 2007. OAUTHC is a multi-center facility that serves as the lead referral center in Osun State and neighbouring Ondo and Ekiti States with a combined 2006 population of over ten million [26]. The hospital has two tertiary units – Wesley Guild Hospital, Ilesa and Ife Hospital Unit, Ile-Ife. The study was conducted simultaneously at the two tertiary units; both provide emergency obstetric care and have full complement of maternal health and neonatal care infrastructures and service providers including obstetricians, anesthesiologists, neonatologists, laboratory scientists and nurse/midwives. While the study period was one-year, there were periods during which the study was interrupted for instance during industrial crises by health workers and so on ; as such the rate for miss reported in this study was for an interrupted six-month period. The study protocol was approved by Ethics and Research Committee of the hospital. Informed consent was obtained from the study participants and participation was voluntary.

### Study population, sample size and selection

The study population consisted of pregnant women who sought care at the hospitals during antenatal (third trimester), intrapartum or within 42 days after delivery. A maternal near miss was defined as any woman who experienced a life-threatening complication and who nearly died but for the hospital care she received. The operational definitions for the near miss were based on the disease-specific criteria described by Filippi et al. [27] which was also utilized by Oladapo et al. [28] in a study on near misses in Sagamu, Nigeria. These are (i). Haemorrhage (leading to shock, emergency hysterectomy, coagulation defects, and/or blood transfusion of 2 or more litres of blood); (ii). Hypertensive disorders in pregnancy - eclampsia and severe pre-eclampsia with clinical or laboratory indication for termination of pregnancy to save the woman’s life (iii). Dystocia - uterine rupture and impending rupture e.g. prolonged obstructed labour with previous caesarian section); (iv). Infection - septicaemia from any cause; (v). Severe anaemia: (hemoglobin <6 g/dl). For every near miss case, four unmatched hospital controls were selected within a defined time limit of 48 hours around the near miss event.

Near misses events were identified by resident doctors in labour ward according to the above-mentioned criteria. The women who survived were interviewed using structured pre-tested questionnaires which were administered by trained research assistants who were all medical personnel (Additional file 1). In addition, pertinent information was also abstracted from their medical records (case notes, operation notes, nurses’ reports and discharge summaries) of respondents. Also, because of the prospective nature of the study, the health and well being of the newborn in the immediate post partum period was also assessed. The perinatal outcomes of the infants of the respondents were assessed which included gestational age at birth, stillbirth or live-birth, infants condition at 1,5 and 10 minutes using APGAR score, birth weight. However some of these parameters could not be considered for women who were delivered outside the health facility and brought in into the hospital in the post-delivery period as cases of emergency obstetric care.

The number of near misses required was estimated using Epi-info version 6 for sample determination of two unequal groups of an unmatched case-control study. The parameters for the calculation were: prevalence of near misses of 17% (based on previous work in Nigeria), [28] power of 80% at 5% statistical significance level. The minimum sample size required were: 64 near miss cases to 256 controls. Pregnant women meeting the study criteria were sequentially recruited as they presented within the study period, and control recruited in similar fashion.

### Statistical analysis

Statistical analysis was performed using STATA version 8. Univariate analysis was carried out to characterize the near miss cases. The differences in the proportion of the characteristics of near misses and controls were compared using chi-square test. Risk factors were assessed using bivariate logistic regression and the Odds Ratio and 95% confidence interval are reported. For the multivariate analysis; the dependent variable was near miss and the independent factors derived from the conceptual framework – background, proximate and clinical factors which were fitted into the models one after the other. Factors included in the model were those found significant at the bivariate level. The three types of models in the multivariate analysis: Model A had only the background characteristics of the respondents as independent variable, while Model B included proximate determinants in addition, while Model C further added clinical factors as part of the independent variables. Perinatal outcomes were also examined for significant association using both chi-square test and bivariate logistic regression. The dependent variable was still near miss and the independent factors were the different perinatal outcomes.

## Results

### Socio-demographic, reproductive health and clinical characteristics

The mean age was 28.6 years (6) and 29.8 years (5) among near misses and control respectively (p=0.032). As shown in Table 1 the age distribution of the two groups was significantly different (p =0.046). Whereas in the near miss group, age group 20 -29 years had the largest proportion of the study participants (54.7%) in the control population age group 30 -39 years had the largest proportion (48.0%) The near miss also had a greater proportion of mothers aged 40 years and above compared to the control group (5.3% versus 2.7% p=0.046). The near-miss group was significantly different from the control group in terms of having less proportion of married women (73.3% versus 93.3%, p<0.001), those living with their spouse (74.4% versus 87.3%, p=0.006), and those whose husbands had post-secondary education (44.0% versus 49.3%, p=0.005). There was however, no significant difference in the groups in terms of the respondents gravidity (p=0.914), parity (p=0.887) level of maternal education (p=0.633) and their religious affiliation (p=0.815).

**Table 1:** Comparison of demographic and reproductive health characteristics of near miss cases and controls

**Characteristics**	**Near miss** **n=75(%)**	**Controls** **n=300(%)**	**Chi-square**	**p-value**
**Age (Years)**				
< 20	4(5.3)	5(1.7)	7.983	0.046
20 - 29	41(54.7)	141(47.0)		
30 - 39	26(34.7)	146(48.0)		
40+	4(5.3)	8(2.7)		
**Maternal education**				
Primary or less	12(16.0)	38(12.7)	0.916	0.633
Secondary	30(40.0)	114(38.0)		
Post Secondary	33(44.0)	148(49.3)		
**Husband’s education**				
Primary or less	16(21.3)	25(8.3)	10.635	0.005
Secondary	25(33.3)	107(35.7)		
Post Secondary	34(45.2)	168(56.0)		
**Religion**				
Christianity	65(86.7)	263(87.7)	0.0545	0.815
Islam	10(13.3)	37(12.3)		
**Marital status**				
Currently				
Married	55(73.3)	274(91.3)	18.064	<0.001
Unmarried	20(26.7)	26(8.7)		
**Living arrangement**				
Lives with	56(74.7)	262(87.3)	7.4686	0.006
spouse	19(25.3)	38(12.7)		
Lives separately				
**Gravidity**				
1-2	46(61.3)	182(60.7)	0.1805	0.914
3-4	19(25.3)	82(27.3)		
5 & above	10(13.4)	36(12.0)		
**Parity**				
1-2	50(66.7)	193(64.3)	0.2393	0.887
3-4	19(25.3)	78(26.0)		
5 & above	6 (8.0)	29(9.7)		
**Contraceptive use prior to conception**				
Use	14(18.7)	69(23.0)	0.6537	0.419
Non Use	61(81.3)	231(77.0)		
**Booking status**				
At OAUTHC	21(29.3)	219(73.0)	59.487	<0.001
Unbooked at OAUTHC	53(70.7)	81(27.0)		
**Referral status**				
Referred	47(62.7)	63(21.0)	0.903	0.342
Not referred	7(9.3)	17(5.6)		

In terms of reproductive health characteristics, the booking status was significantly different between the two groups of mothers, with a majority of the near misses (70.7%) not obtaining antenatal care at the tertiary facility compared with the controls (27.0%). There was no significant difference in the parity (p=0.887), contraceptive use prior to conception (p=0.419) and referral status (p=0.342)

The incidence rate of near miss over an uninterrupted six month period was 12% (42 near misses out of a total of 382 deliveries). Majority of the near miss morbidities resulted from severe haemorrhage (41.3%) and hypertensive disorders in pregnancy (37.3%) (Table 2). Near misses with prolonged obstructed labour (23%) had other co-morbidities like septicaemia 4 (5.3%), stillbirth 4(5.3%) and ruptured uterus 2(2.7%). Septicaemia which occurred in 18.6% of cases resulted from puerperal sepsis 11(14.6%) and chorioamnionitis 3(4.0%). Severe malaria was the commonest cause (7 out of 11 cases) of severe anaemia which occurred in 14.6% of the cases.

**Table 2:** Distribution of near-miss cases by clinical conditions

**Causes of near-miss**	**Near-miss cases due to the specific conditions (n=75)**	
	**Frequency**	**%**
**Haemorrrhage**	**34**	**45.3**
Antepartum haemorrhage	6	8.0
Post-partum haemorrhage	28	37.3
Proportion in shock	23	30.7
Mean units of blood transfused	3 [2-10]	**-**
**Hypertensive disorders of pregnancy**	**28**	**37.3**
Severe pre-eclampsia	19	25.3
Eclampsia	9	12.0
**Dystocia**	**18**	**23.0**
Proportion with co-morbidities	10	13.3
Still birth	4	5.3%
Septicaemia/Septic shock	4	5.3%
Ruptured Uterus	2	2.7%
**Septicemia**	**14**	**18.6**
Puerperal sepsis	11	14.6
chorioamnomitis	3	4.0
**Severe anaemia**	**11**	**14.5**
Malaria	7	9.3
others	4	5.2

### Determinants of near misses

The result of the binary logistic regression analysis for the determinants of near miss maternal morbidity is presented in Table 3. In model A, which focuses on socio-demographic factors alone, marital status was the only significant factor for near miss; the odds of a near miss was about three times in the unmarried compared to those currently married (OR=3.09; 95% CI: 1.49 -6.38).

**Table 3:** Binary logistic regression analysis of the determinants of near miss

	**Model A**	**Model B**	**Model C**
**Variables**	**Odd’s ratio (95%CI)**	**Odd’s ratio (95%CI)**	**Odd’s ratio (95%CI)**
**Background variables**			
**Age**			
<20	1.43 (0.33 – 6.20)	1.06(0.22 – 5.10)	0.82 (0.16 – 4.35)
20 – 34	1.00	1.00	1.00
35+	1.02 (0.52 – 1.97)	1.07 (0.51 – 2.31)	1.12 (0.51 – 2.49)
**Husband’s education**			
Secondary or less	1.41 (0.84 – 2.38)	0.97 (0.53 – 1.76)	0.97 (0.52 – 1.81)
Post Secondary	1.00	1.00	1.00
**Marital status**			
Currently married	1.00	1.00	1.00
Unmarried	**3.09 (1.5 – 6.38)**	2.34(0.98– 5.60)	2.00 (0.79 – 5.06)
**Living arrangement**			
Lives with spouse	1.00	1.00	1.00
Lives separately	1.53 (0.76 – 3.09)	1.07 (0.45 – 2.56)	1.2 (0.49 – 2.95)
**Proximate determinants**			
**Wantedness of pregnancy**	-	0.63 (0.30 – 1.35)	0.66 (0.30 – 1.46)
**Antenatal care**		**0.21 (0.11 – 0.41)**	**0.19 (0.09 – 0.37)**
**Knowledge of complications**	-	**0.53 (0.27 – 1.02)**	**0.47 (0.24 – 0.94)**
**Male support**	-	0.98 (0.32 – 2.97)	0.98 (0.31 – 3.09)
**Phase one delay**	-	**2.07 (1.03 – 4.17)**	**2.10 (1.04 – 4.27)**
**Phase two delay**	-	0.91 (0.41 – 1.99)	0.96 (0.44 – 2.11)
**Chronic hypertension**	-	**9.3 (2.77 – 31.34)**	**6.85(1.96 – 23.9)**
**Clinical variables**			
**Foetal presentation**			
Cephalic	-	-	1.00
Malpresentation			**0.16 (0.40 – 0.67)**
**BP in Labour**			
<140/90	-	-	1.00
>140/90			1.23 (0.65 – 0.2.32)
**Mode of delivery**			
SVD	-	-	1.00
Emergency C/S			**3.72(0.93 – 14.9)**
Others			2.55 (1.34 – 4.83)

Model B included both background and proximate determinants as independent variables. In this model, where as none of the socio-demographic factors showed any statistical significance, some proximate factors showed significant association with near miss event. On the one hand, a prior history of chronic hypertension [OR= 9.3; 95% CI: (2.77 – 31.34)] and having experienced a phase one delay [OR=2.07; 95% CI (1.03 – 4.17)] increased the odds of experiencing a near miss event. Antenatal care attendance at a tertiary facility [OR=0.19; 95% CI (0.09 -0.38)] was protective of a near miss event, reducing the risk by 5 times. Knowledge of pregnancy complications also had a borderline significant relationship with near miss, reducing the risk by half [OR=0.53; 95% CI (0.27 – 1.02)].

In Model C, containing socio-demographic factors, proximate determinants and clinical variables, the results in Model B were sustained. In addition, while emergency caesarian section had borderline statistical significance [OR=3.72; 95% CI (0.93 – 14.9)], assisted vaginal delivery increased the odds of a near miss event significantly [OR=2.55; 95% CI (1.34 – 4.83)].

### Perinatal outcomes

The findings regarding perinatal outcomes among the near misses and controls are presented in Table 4. The frequency of still birth was significantly higher among near misses compared to controls (28.4% versus 4.8%, P<0.001). Infants of women who had experienced life-threatening complications also had comparably higher proportion of severe birth asphyxia (22.2% versus 6.0% p< 0.001). The proportion of low-birth weight infants (<2500 g) was higher among near misses compared to controls (44.4% versus 13.5% p<0.001). The logistic regression analysis also revealed significant associations between near miss and stillbirth [OR=5.40; 95% CI (2.18 – 13.40)], low birth weight [OR=3.38; 95% CI (1.61 – 7.06)] and post mature pregnancy [OR=3.24; 95% CI (1.51 – 6.97)].

**Table 4:** Perinatal outcomes of respondents

**Perinatal outcomes**	**Near misses**	**Controls**	**Odd’s ratio 95% CI**	**p-value**
**Pregnancy outcome**				
Live birth (RC)	53(71.6)	275(95.2)	1.00	-
Still birth	21(28.4)	14(4.8)	5.40 (2.18 - 13.40)	<0.001
**Apgar score**^**1**^				
Severe asphyxia	10(22.2)	16 (6.0)	-	
Mild/Moderate asphyxia	15(33.3)	79(28.2)	-	
Good (RC)	21(44.0)	175(65.8)	-	
**Birth weight**				
LBW (<2500 g)	28(44.4)	38(13.5)	3.38 (0.79 -3.68)	0.001
Normal (2500 g – 4000 g)	33(52.4)	228(80.9)	1.00	-
(RC)	2(3.2)	16(5.7)	0.77(0.16 -3.61)	0.736
Macrosomia [>4000 g]			-	
**Maturity at birth**				
Prematurity (<38 weeks)	31(41.3)	66(22.0)	1.7(0.79 -3.68)	0.175
Term (38 – 40 weeks)	24(32.0)	179(59.7)	1.00	-
Post mature (>40 weeks)	18(24.0)	54(18.0)	3.24(1.51 -6.97)	0.002

1. Apgar score was omitted from the logistic regression model due significant missing data on Apgar score from infants who had experienced still-birth in whom this could not be assessed.

## Discussion

A sustained commitment to maternal health issues in Nigeria is vital to the attainment of Millennium Development Goal 5 globally. This is because Nigeria, with an estimated current population of over 160 million, is the most populous country in Africa as well as the second largest contributor of maternal deaths globally. This prospective case control study on the determinants and perinatal outcomes of near miss maternal morbidity was conducted in South Western Nigeria. A prospective approach has an advantage over a retrospective study in investigating etiologic relationships as it deals with incident rather than prevalent cases [29]. The incidence of near misses in this study was 12%. While this figure falls within the range of 1.17 – 23.8% [27] reported by Fillipi et al. in three West African countries (Benin, Cote d’Ivoire and Morocco), it is slightly lower than the estimate of 17% in an earlier Nigerian study carried out in Sagamu. Whereas both locations are in south-west Nigeria, which is populated mostly by Yorubas, their socio-demographic mix differed somehow. Sagamu, for example, has a higher proportion of Hausa – whose maternal health seeking behaviour differed significantly from the Yorubas. As national surveys such as the National Demographic and Health Surveys [26] and National HIV/AIDS and Reproductive health Survey (NARHS) [30] have shown, the maternal and child health seeking behaviour and indices are much better in areas predominantly occupied by Yorubas (South-West-political zone) compared to those predominantly inhabited by Hausas (North-East and North-West geo-political zones). Thus, the population mix in terms of ethnicity and the associated different maternal health-seeking behaviours may account for the differences in the estimates recorded for the two studies. It is important to note that the estimates from both this study and that of Sagamu are not likely to be representative of the true incidence of the near miss for the entire country because there are wide geo-political variations in health indices in Nigeria and the south west has the best maternal health indices compared to other geo-political regions. In addition, fact that both studies are carried out in tertiary facilities also have implications for the representativeness of the figures for the entire countries.

Haemorrhage and hypertensive disorders in pregnancy were the two leading causes of near misses in this study; this is consistent with earlier studies in most part of the world [10,20,27,31]. These obstetric events are also the leading causes of maternal death in Nigeria [32] and most other developing countries. Severe anaemia attributable to severe malaria contributed considerably to the near miss burden in this study. This may be explained by the holoendemicity of malaria in the study area and it also emphasizes the importance malaria prevention in pregnancy through the use of long lasting insecticide treated bed nets, intermittent preventive treatment and prompt case management of malaria in pregnancy.

In this study, chronic hypertension has the strongest association as a risk factor for near misses with a seven fold increase in risk. Hypertension and diabetes have been predictors of near misses in the United Kingdom [10]. Chronic hypertension considerably increases the risk of complications in pregnancy like superimposed pre-eclampsia, placental abruption, intra-uterine growth retardation and preterm delivery among others [33]. Therefore, chronic hypertension in pregnancy may be a risk marker and a premise for referral to a higher facility. Pregnant women with chronic hypertension (or any other medical condition) need to be carefully monitored and managed during pregnancy in order to prevent various potential complications. In addition such women must be managed in a facility that can provide emergency essential obstetric and neonatal care. The increase in the occurrence of chronic diseases in developing countries [34,35] and its relationship with pregnancy outcomes require further research.

Phase one delay, which is the delay in making the decision to seek care, was also an important risk factor for near miss in this study. Delays in accessing obstetric care during life-threatening complications is a major reason for poor maternal health outcomes in developing countries [35]. In our study about three-fifths (60.0%) of the near miss cases experienced either phase one and phase two delays (delay in reaching health facility) which resulted from underestimating the severity of various pregnancy-related conditions, lack of available transport particularly for problems occurring in the night as well as first seeking care from a facility that is ill-equipped to provide emergency obstetric care. Poor knowledge of risk associated with various pregnancy warning signs as well as the failure to identify health facilities well equipped for the provision of emergency obstetric services may play a major part in care-seeking decisions. Our findings that antenatal care attendance and knowledge of complications have significant protective effect against near-miss are relevant in this regard. These findings have significant implications for interventions alongside the various phases of delay are modifiable through appropriate interventions. Antenatal care offers a unique platform for the provision of cost effective health interventions which will ensure healthy outcomes for pregnant women [36]. These include health promotion and preventive services; early detection and treatment of complications and existing diseases; birth preparedness and complication readiness together with promoting male participation. All these are the essential ingredients of quality antenatal care.

The determinants most amenable to change are those linked to obstetric interventions for instance, emergency caesarean section (odds ratio 3.72) and assisted vaginal delivery (odd ratio 2.55). Waterstone et al. also found a strong association between emergency caesarean section and near misses in the United Kingdom. The increased odds of near miss may be associated with the outcome or survival of a near miss rather than being a risk factor due to the temporal sequence of the events. This is because such treatment modalities are employed after the occurrence of a complication and not vice-versa. This notwithstanding, this increased risk associated with emergency caesarean section may be related to the aversion of women and their family members towards caesarian delivery in developing countries such that even in event of a complication women are reluctant to access care until their conditions become life threatening.

When socio-demographic factors alone were considered as a group, being unmarried was the only significant determinant among the socio-demographic characteristics (odd ratio 3.09). It however, became insignificant after adjustment for the proximate risk factors. Marital status, although not amenable to change, may bring to light the issue of male involvement in obstetric care. Adewuyi et al. in an interventional study in south western Nigeria demonstrated that women who lacked male support were more likely to require emergency obstetric care [37].

Perinatal outcomes are important indicators of maternal and newborn health care. In this study, stillbirth (odds ratio 7.15) low birth weight (odds ratio 3.38), and post mature pregnancy (odds ratio 3.24), were strongly associated with near misses. Although several studies have reported the link between maternal morbidity and adverse perinatal outcome very few have described their relationship to near miss maternal morbidity. An example of the latter was the study of Fillipi and her colleagues in Burkina Faso where they also demonstrated a significant association between near misses and stillbirth [18]. Considerably, the factors that increase the risk of adverse maternal and perinatal outcomes are quite similar for instance inadequate care during pregnancy, inappropriate management of complications, lack of newborn care and so on. Therefore, efforts directed at ensuring maternal health will have a multiplier effect which will invariably impact on the reduction of child mortality. This becomes highly significant in the light of the attainment of the Millennium Development Goals particularly MDG4 AND 5.

A challenge in a study of this nature is the number of ‘cases’ as near miss is a rare event. However, this was addressed by using a high case to control ratio (1:4) thus increasing the statistical power of the study. In addition, attempts were made to minimize some of the problems associated with a case control design. For instance, recall and misclassification biases were lessened by using incident rather than prevalent cases as well as employing validated operational definitions in the selection of cases. Lastly, although the study was conducted over a one year period there were times the study was discontinued due to circumstances beyond the control of the researchers particularly industrial action by health workers; which interrupted the study for a period of time. Hence the near miss rate in this study was limited to an uninterrupted six-month period. Studies on determinants and perinatal outcomes of near miss that address these limitations need to be performed in future, preferably prospective multicenter study carried out over a period of two years or more to generate more stable estimates. Also, near-miss studies need to be conducted in other parts of the country to produce a more comprehensive national picture of the near miss morbidity for Nigeria. Finally, it is imperative that findings from this study be used to inform interventions as Nigeria continues to strive towards achieving the fourth and fifth Millennium Development Goal.

## Conclusions

In summary, the analysis of near misses has evolved as a useful tool in the investigation of maternal ill health especially in life-threatening situations. The key determinants of near miss in this study were: phase one delay, history of chronic hypertension, emergency caesarean section and assisted vaginal delivery. Quality antenatal care and the knowledge of complications were found protective of a near miss event. Many of these are amenable by appropriate public and medical interventions. Importantly, chronic hypertension was the strongest determinant of near miss in this study. Therefore with the increasing burden chronic diseases globally, their interaction with pregnancy and its outcome requires careful research in the future. In addition, near misses are strongly associated with adverse perinatal outcomes which imply that efforts directed at maternal health will inadvertently lead to reduction of child mortality.

## Competing interests

The authors declare that they have no completing interest.

## Authors’ contributions

IAA and AAO designed the study. IAA conducted the study under the supervision of AAO and AOF. IAA analyzed the data and wrote the initial draft of the manuscript. AAO and AOF reviewed and critically revised the manuscript. All authors read and approved the final manuscript.

## Pre-publication history

The pre-publication history for this paper can be accessed here:

http://www.biomedcentral.com/1471-2393/13/93/prepub

## Supplementary Material

Additional file 1 Determinants and Outcome of Near Miss Maternal Morbidity in a Tertiary Hospital in South West, Nigeria: Data Collection Instrument. Click here for file
