# Supplementary material for: Incidence, determinants and perinatal outcomes of near miss maternal morbidity in Ile-Ife Nigeria: a prospective case control study
Source: BMC Pregnancy Childbirth. 2013 Apr 15;13:93. doi: 10.1186/1471-2393-13-93 (PMC3651395; doi:10.1186/1471-2393-13-93)
Supplement: Additional file 1 — Determinants and Outcome of Near Miss Maternal Morbidity in a Tertiary Hospital in South West, Nigeria: Data Collection Instrument. [file 1471-2393-13-93-S1.docx]

**APPENDIX 1**

**Determinants and Outcome of Near Miss Maternal Morbidity in a Tertiary Hospital in South West, Nigeria.**

**QUESTIONNAIRE**

Dear Respondent,

This is a study on the occurrence and outcome of life threatening complications among women during pregnancy, delivery and immediately after delivery. The information to be elicited with this questionnaire is strictly for academic purposes. All information provided will be kept confidential. We humbly seek your support and cooperation.

Thank you for your anticipated cooperation.

Yours sincerely,

Dr. I.A Adeoye

Investigator

HOSPITAL NO: …………………..

SERIAL NO ……………………….

| 1 | How old were you as at your last birthday. | | | | | | | | | ------------------------- | | | |  | | |  | |  |  |
| --- | --- | --- | --- | --- | --- | --- | --- | --- | --- | --- | --- | --- | --- | --- | --- | --- | --- | --- | --- | --- |
| 2 | What is your highest level of academic qualification | | | | | | | | | None  Primary  Secondary School  NCE / Polytechnic  University degree  High degree | | | | 0  1  2  3  4  5 | | |  | |  |  |
| 3 | What is your husband’s/partner’s highest academic qualification | | | | | | | | | None  Primary  Secondary School  NEC/Polytechnic  University  Higher degree | | | | 0  1  2  3  4  5 | | |  | |  |  |
| 4 | Religion of Respondent | | | | | | | | | Christianity  Islam  Traditional  Others (Specify) | | | | 1  2  3  -------------- | | |  | |  |  |
| 5 | Are you currently married or you are just living in with a partner | | | | | | | | | Never married  Currently married  Cohabiting  Separated  Others specify | | | | 1  2  3  4 | | |  | |  |  |
| 6 | Do you live in the same house with your husband / partner | | | | | | | | | live together  live separately | | | | 1  2 | | |  | |  |  |
| 7 | What type of marriage | | | | | | | | | Monogamous  Polygamous | | | | 1  2 | | |  | |  |  |
| 8 | What is your occupation? | | | | | | | | |  | | | |  | | |  | |  |  |
| 9 | What is your husband’s occupation | | | | | | | | |  | | | |  | | |  | |  |  |
| 10 | What is your usual place of residence | | | | | | | | |  | | | |  | | |  | |  |  |
| 11 | How far is your usual place of usual place of residence to this hospital | | | | | | | | | …………………km | | | |  | | |  | |  |  |
|  | How far is your usual place of usual place of residence to the hospital you planned to deliver? | | | | | | | | | …………………km | | | |  | | |  | |  |  |
|  | What is your monthly income? | | | | | | | | | …………………. | | | |  | | |  | |  |  |
| **Family planning (Wanted ness of pregnancy)** | | | | | | | | | | | | | |  | | |  | |  |  |
| 12 | | At the time you became pregnant; did you want to become pregnant then or later or you did not want any more children? | | | | Then  Later  Not at all | | | | | | | | 1  2  3 | | |  | |  |  |
| 13 | | Prior to your getting pregnant did you use any method to delay or avoid pregnancy | | | | Yes  No | | | | | | | | 1  2 | | |  | |  |  |
|  | | **Past Obstetric History** | | | |  | | | | | | | |  | | | | |  |  |
| 14 | | Total number of previous pregnancies | | | | No of Life Births  Full term  Preterm  No of Induced abortion  Spontaneous1^st^ trimesters miscarriage  Spontaneous 2^nd^ trimester miscarriage  No of Ectopic prenancy  No of molar pregnancies  No of multiple pregnancy  No of Stillbirth  No of Neonatal deaths  Total | | | | | | | | ………………..  ………………..  ………………..  ………………..  …………………  …………………  …………………  …………………  …………………  …………………  _____________ | | | | |  |  |
| 15.1 | | What were the Complications you experienced in your previous pregnancies? (DON’T PROMPT) | | | | Previous Pregnancy induced hypertension  Previous Ante partum haemorrhage  Previous Post partum haemorrhage  Prolong Labour  Previous perinatal deaths  (*Still birth / fetal death*).  Previous Premature rupture of membranes  Preterm labour / delivery  Low birth weight HUGR  Congenital abnormality (Gross)  Puerperal Sepsis  Others specify …………………  …………………………………..  ………………………………….. | | | | | | | | A  B  C  D  E  F  G  H  I  J  K | | |  | |  |  |
| 15.2 | | What are the danger signs in pregnancy (MENTION ALL YOU KNOW) | | | | Spotting/bleeding  Swollen legs  Leaking of water from vagina  Fever  Severe headache  Lack of blood  Offensive/yellowish vagina  Discharge  Othersspecify…………………………………………………........................ | | | | | | | | A  B  C  D  E  F  G  H  I  J | | |  | |  |  |
|  | | Did your husband support you during pregnancy? | | | | Yes  No | | | | | | | | 1  2 | | |  | |  |  |
|  | | In what ways did he support (tick all mentioned)  DO NOT PROMPT | | | | Followed you to the place of delivery  Providing money  Showed affection  Encouraged you to take your drugs  Took care of the children  Reminded you of your clinic days  Others specify………………………………………………………………………………………. | | | | | | | | 1  2  3  4  5  7  8 | | |  | |  |  |
| 15.3 | | Who in your family usually has the final say on the following decisions?   \|  \| Wife \| Husband \| Couple \| In laws \| Your parents \| Friends \| Others specify \| \| --- \| --- \| --- \| --- \| --- \| --- \| --- \| --- \| \| Your own health care \|  \|  \|  \|  \|  \|  \|  \| \| Your care in an emergency situation \|  \|  \|  \|  \|  \|  \|  \| \| Children’s health care \|  \|  \|  \|  \|  \|  \|  \| \| Children’s education \|  \|  \|  \|  \|  \|  \|  \| \| Making large household purchases e.g land \|  \|  \|  \|  \|  \|  \|  \| \| Making household purchases for daily needs \|  \|  \|  \|  \|  \|  \|  \| \| How the money your earn is spent \|  \|  \|  \|  \|  \|  \|  \| | | | | | | | | | | | | | | | | |  |  |
| 15.4 | | Were there times/ or was there a time you were beaten or hit in this pregnancy for any of the following reasons?   \|  \| YES \| NO \| DONT KNOW \| \| --- \| --- \| --- \| --- \| \| For going out without telling him \|  \|  \|  \| \| For neglecting the children \|  \|  \|  \| \| For arguing with him \|  \|  \|  \| \| For refusing to have sex with him \|  \|  \|  \| \| For burning the food \|  \|  \|  \| \| For not cooking his food on time? \|  \|  \|  \| \|  \|  \|  \|  \| | | | | | | | | | | | | | | | | |  |  |
| 16 | | **Obstetric History** | | | | | | | | | | | | | | | | |  |  |
| **Birth Order** | | | | Date of Birth | Place of birth | | Birth Attendant | | | | Pregnancy Outcome | | | Complications experienced. | | | | |  |  |
|  | | | |  |  | |  | | | |  | | |  |  |  |  |  |  |  |
|  | | | |  |  | |  | | | |  | | |  |  |  |  |  |  |  |
|  | | | |  |  | |  | | | |  | | |  |  |  |  |  |  |  |
|  | | | |  |  | |  | | | |  | | |  |  |  |  |  |  |  |
|  | | | |  |  | |  | | | |  | | |  |  |  |  |  |  |  |
| 17  17.1  17.2  17.3  17.4  17.5 | | | Gravidity  Parity  Height  Genotype.  HIV/AIDS Status | | | | | | ------------------------------------------------------------------------------------------------------------------------------------------------------------------------------------------------------------- | | | | |  | | | |  |  |  |
| 18  18.1  18.2  18.3  18.4  18.5  18.6 | | | **Past Medical History**  Hyper tension  Diabetes  Sickle cell Anaemia  HIV/AIDS  Asthma  Others Specify | | | | | | Yes  No  Yes  No  Yes  No  Yes  No  Yes  No  …………………………………..  ………………………………….. | | | | | 1  2  1  2  1  2  1  2  1  2 | |  | | |  |  |
|  | | | **HISTORY OF INDEX PREGNANCY** | | | | | |  | | | | |  | |  | | |  |  |
| 19  20 | | | Last Menstrual Period (D/M/Y)  Expected Date of Delivery (D/M/Y)  Calculated  USS | | | | | | ……………………………..  …………………………………  ……………………………….. | | | | |  | |  | | |  |  |
| 21 | | | Actual Date of Delivery | | | | | | ………………………………. | | | | |  | |  | | |  |  |
| 22 | | | Gestational age at delivery | | | | | |  | | | | |  | |  | | |  |  |
| 23 | | | Number of babies delivered | | | | | |  | | | | |  | |  | | |  |  |
| 24 | | | Interpregnancy Interval | | | | | |  | | | | |  | |  | | |  |  |
|  | | | **ANTENATAL CARE (ANC)** | | | | | |  | | | | |  | |  | | |  |  |
| 25 | | | Did you receive antenatal care in this pregnancy | | | | | | Yes  No | | | | | 1  2 | | If no, skip to 34 | | |  |  |
| 26 | | | If Yes, Where did you receive ANC | | | | | | OAUTHC (BOOKED)  Else where?  Specify………………………………………………………….. | | | | | 1  2 | |  | | |  |  |
| 27 | | | How many weeks pregnancy were you when you first received antenatal care for this pregnancy | | | | | | ………………………………… | | | | |  | |  | | |  |  |
| 28 | | | How many times did you receive ANC during this pregnancy (Total No of visit) | | | | | | ……………………………… | | | | |  | |  | | |  |  |
| 29 | | | Were you told about the signs of pregnancy complications at  Booking Visit | | | | | | Yes  No | | | | | 1  2 | |  | | |  |  |
| 30  30.1  30.2  30.3  30.4  30.5  30.6 | | | Which services did you receive during the antenatal period? | | | | | | Tetanus toxoid  Haematinics  Anti Malarias  Antiretroviral  Antibiotics  Others specify……………………………………………………………………………………………… | | | | | 1  2  3  4  5  6 | |  | | |  |  |
| 31 | | | What was your Booking Blood Pressure? | | | | | |  | | | | |  | |  | | |  |  |
| 32 | | | What was your booking weight? | | | | | |  | | | | |  | |  | | |  |  |
| 33  33.01  33.02  33.03  33.04  33.05  33.06  33.07  33.08  33.09  33.10  33.11 | | | What were the maternal problems noted at Booking | | | | | | Elderly Primigravida  Grand Multi -gravida  Teenage Pregnancy  Unmarried  Diabetes Mellitus  Hypertension  Sickle cell disease  HIV/AIDS  Epilepsy  Previous C/S  Ante partum Haemorhage  Others Specify…………………………………………………………… | | | | | A  B  C  D  E  F  G  H  I  J  K  L | |  | | |  |  |
| 34 | | | If Unbooked, Why did you come to OAUTHC | | | | | | Was referred  ANC outside was suboptimal  Feet like  Other Specify | | | | | 1  2  3  4 | |  | | |  |  |
| 35 | | | If 1, at gestational age were you referred? | | | | | | …………………………………. | | | | |  | |  | | |  |  |
| 36 | | | From which facility were you referred? | | | | | | Primary Health Centers  Private maternity  Government maternity  Private hospital  Government hospital  Mission homes  TBAs  Mission hospitals  Others specify …………………...  …………………………………... | | | | | 1  2  3  4  5  6  7  8  9 | |  | | |  |  |
| 37 | | | What was the indication for referring you to this hospital | | | | | |  | | | | |  | |  | | |  |  |
| 38 | | | Did you have any antenatal admission | | | | | | Yes  No | | | | | 1  2 | | If no, skip to 42 | | |  |  |
| 39 | | | If Yes, at what gestational age then | | | | | | ………………………………… | | | | |  | |  | | |  |  |
| 40 | | | What was the reason/s for the antenatal admission | | | | | | Hyper emesis  Threatened Abortion  Antepartun Haemorhage  Diabetes  Malaria  Urinary Tract Infection  Hepatitis  PROM Fetal problem Specify  Anaemia  Hypentension  Others specify | | | | | A  B  C  D  E  F  G  H  I  J  K  L | |  | | |  |  |
| 41 | | | How long was the antenatal admission | | | | | | …………………….. | | | | |  | |  | | |  |  |
| 42 | | | **Birth preparedness** – What preparations did you make towards the birth of your child? | | | | | | | | | | | | | | | |  |  |
| 42.1 | | | Did you have special savings to pay for expenses incurred? (i.e open a new savings account? | | | | | | Yes  No | | | | 1  2 | |  | | | |  |  |
| 42.2 | | | Did you identify a particular health facility where you intended to have your baby? | | | | | | Yes  No | | | | 1  2 | |  | | | |  |  |
| 42.3 | | | Did you identify those that will accompany or stay with you in the hospital | | | | | | Yes  No | | | | 1  2 | |  | | | |  |  |
| 42.4 | | | Did you identify those that will look after you other children at home when in the hospital? | | | | | | Yes  No | | | | 1  2 | |  | | | |  |  |
| 42.5 | | | Did you make any arrangement for a means of transportation to take you to the hospital | | | | | | Yes  No | | | | 1  2 | |  | | | |  |  |
| 42.6 | | | Did you buy and store materials needed during and after your delivery | | | | | | Yes  No | | | | 1  2 | |  | | | |  |  |
| 42.7 | | | Were you aware of the causes, signs and symptoms of various complications before, during and after pregnancy? | | | | | | Yes  No | | | | 1  2 | |  | | | |  |  |
| 42.8 | | | Did you know how to call for help or when and how to get to the hospital before the complications became serious? | | | | | | Yes  No | | | | 1  2 | |  | | | |  |  |
|  | | | **DELAYS** | | | | | |  | | | |  | |  | | | |  |  |
| 43 | | | Who was involved in making the initial decision that you should come to the hospital | | | | | | Self  Husband  Couple  Mother in law  Relatives  Friends  Others | | | |  | |  | | | |  |  |
| 44 | | | Who was involved in making the final decision that you should come to the hospital | | | | | | Self  Husband  Couple  Mother in law  Relatives  Friends  Others | | | |  | |  | | | |  |  |
| 45 | | | Was there any delay in making a decision to go to the hospital | | | | | | Yes  No | | | | 1  2 | | If no, skip to 47 | | | |  |  |
| 46 | | | If yes, what were the reasons for the delay? | | | | | | Underestimated severity of condition.  Did not realize the was a problem  Bad experience with health system  Essential people in decision making not around  Disagreement in decision making  Believed that God was in control  Others Specify …………………………………………………………………… | | | | 1  2  3  4  5  6  7 | |  | | | |  |  |
| 47 | | | What symptoms prompted you to come to the hospital | | | | | |  | | | |  | |  | | | |  |  |
| 48 | | | Once the decision was made to go to the hospital did you go straight away? | | | | | | Yes  No | | | | 1  2 | | If yes, skip to 51 | | | |  |  |
| 49 | | | Why not? | | | | | | Lack of Money  Lack of transport | | | | 1  2 | |  | | | |  |  |
| 50 | | | How long was the delay? | | | | | | ……………….(mins/hours/days) | | | |  | |  | | | |  |  |
| 51 | | | Was it difficult to find the funds to come to the hospital? | | | | | |  | | | |  | |  | | | |  |  |
| 52 | | | Where did the funds come from for you to come to the hospital (i.e who paid/or who is going to pay) | | | | | |  | | | |  | |  | | | |  |  |
| 53 | | | How did you get to the hospital | | | | | | Personal car  Personal motorbike  Ambulance  Public transport(……………………………………………………)  Others specify……………………………………………………………. | | | | 1  2  3  4  5 | |  | | | |  |  |
| 54 | | | How long did it take you to get here | | | | | | …………………….. | | | |  | |  | | | |  |  |
| 55 | | | Did you have any difficulties in transporting yourself to the hospital? | | | | | | Yes  No | | | | 1  2 | | If no, skip to 57 | | | |  |  |
| 56 | | | What were these difficulties? | | | | | | Poor road conditions  Poor vehicle conditions  Lack of available transport  Problem occurred at night  No fuel  No ambulance  Long distance  Had first seek care from another facility  Others specify……………………………………………………………... | | | | 1  2  3  4  5  6  7  8  9 | |  | | | |  |  |
| 57 | | | When you got to the hospital, how long did you wait before you were first seen by a health professional? | | | | | | ……………………………. | | | |  | |  | | | |  |  |
| 58 | | | What time did you get to the hospital? | | | | | | …………………………….. | | | |  | |  | | | |  |  |
| 59 | | | What time were you first seen? | | | | | | …………………………… | | | |  | |  | | | |  |  |
|  | | |  | | | | | |  | | | |  | |  | | | |  |  |
|  | | | **Labour** | | | | | |  | | | |  | |  | | | |  |  |
| 60 | | | Duration of 1^st^ stage (hrs) | | | | | |  | | | |  | |  | | | |  |  |
| 61 | | | Duration of 2^nd^ stage (mins) | | | | | |  | | | |  | |  | | | |  |  |
| 62 | | | Duration of 3^rd^ stage of labour | | | | | |  | | | |  | |  | | | |  |  |
| 63 | | | Fetal presentation labour | | | | | | Cephalic vertex  Breech type  Brow presentation  Face presentation | | | | 1  2  3  4 | |  | | | |  |  |
| 64 | | | What was the Lie in Labour | | | | | | Oblique  Longitudinal  Transverse  Others Specify | | | | 1  2  3  4 | |  | | | |  |  |
| 65  65.1  65.2  65.3  65.4 | | | Vital Signs during labour | | | | | | Blood Pressure  Temperature  Pulse rate  Respiratory Rate | | | | ……..  …………..  ……. | |  | | | |  |  |
| 66 | | | Type of delivery | | | | | | Spontaneous vertex delivery  Assisted breech delivery  Vacuum  Forceps  Elective C/S  Emergency C/S  Induction of labour | | | | 1  2  3  4  5  6  7 | |  | | | |  |  |
| 67 | | | How was the 3^rd^ stage of labour managed? | | | | | | Control cord fraction  Manual Removal  Evacuation  Other Specify | | | | 1  2  3  4 | |  | | | |  |  |
| 68 | | | Who attended the birth? | | | | | | Midwifery Student  Medical Student  Midwife  Resident  Consultant | | | | A  B  C  D  E | |  | | | |  |  |
| 69 | | | What was the indication of the Caesarian Section | | | | | | Prolonged labour  Previous c/s (FTOS)  CPD  Fetal distress  PET/Eclampsia  Fetal malposition  Breech presentation  Antepartum Haemorrhage  Abnormal lie  Cord accident  Chorioamnionitis  Failed induction  Others specify……………………  …………………………………... | | | | 1  2  3  4  5  6  7  8  9  10  11  12  13 | |  | | | |  |  |
| 70 | | | What was the indication of the induction of labour | | | | | | ………………………………………………………………………………………………………………………………………… | | | |  | |  | | | |  |  |
| 71  71.1  71.2 | | | What were the complications experienced during labour | | | | | | Maternal…………………………………  ……………………………………………………………………………………………………………………………………….  Fetal……………………………………………………………………………………………………………………………………………………………………………… | | | | | |  | | | |  |  |
| 72 | | | What was the length of hospital stay? | | | | | Date of admission  Date of Discharge | | | | -------------  -------------- | | |  | | | |  |  |
|  | | | **PREGNANCY OUTCOME** | | | | | |  | | | | |  |  | | | |  |  |
| 73 | | | What was the outcome of this Pregnancy? | | | | | | Live Birth  Still birth before labour  Still birth after labour  Congenital abnormally …..………………..……………  …………………………………..  Others specify……………………  ………………………………….. | | | | | 1  2  3  4 |  | | | |  |  |
| 74 | | | What was the birth weight | | | | | |  | | | | |  |  | | | |  |  |
| 75 | | | What was the Apgar score at? | | | | | | 1 minute ------------------------------  5 minutes -----------------------------  10 minutes --------------------------- | | | | |  |  | | | |  |  |
| 76 | | | Was there any neonatal admission | | | | | | Yes  No | | | | | 1  2 | If no, skip 79 | | | |  |  |
| 77 | | | If yes, what was the indication? | | | | | | ………………………………. | | | | |  |  | | | |  |  |
| 78 | | | What is the case note number | | | | | | ………………………………. | | | | |  |  | | | |  |  |
| 79 | | | What was the perinatal outcome | | | | | | ……………………………….. | | | | |  |  | | | |  |  |
| 80 | | | **Has there been a near miss maternal morbidity in this pregnancy according to the predetermined criteria?** | | | | | | Yes  No | | | | | 1  2 |  | | | |  |  |
|  | | | If yes, continue with the next section and if no, stop here! | | | | | |  | | | | |  |  | | | |  |  |
|  | | | **SEVERE MORDID EVENT** | | | | | |  | | | | |  |  | | | |  |  |
| 81 | | | Date /time of onset of symptoms  Date (D/M/Y)  Time 00:00 | | | | | |  | | | | |  |  | | | |  |  |
| 82 | | | Date and time of diagnosis  Date (D/M/Y)  Time 00:00 | | | | | |  | | | | |  |  | | | |  |  |
| 83 | | | Timing of event | | | | | | Antepartun  Intra - partum  Post partum | | | | | 1  2  3 |  | | | |  |  |
| 84 | | | When exactly did the severe morbid event occur? | | | | | | During admission  Had occurred before arrival | | | | | 1  2 |  | | | |  |  |
| 85 | | | What was the gestational age at occurrence? | | | | | | …………………………… | | | | |  |  | | | |  |  |
|  | | | **SPECIFIC DISEASE CONDITION**  **Preclampsia/Eclampsia** | | | | | |  | | | | |  |  | | | |  |  |
| 86 | | | Was there severe preclampsia/Eclampsia | | | | | | Yes  No | | | | | 1  2 | If no skip 97 | | | |  |  |
| 87 | | | What was the highest blood pressure recorded | | | | | |  | | | | |  |  | | | |  |  |
| 88 | | | What was the highest level of proteinuria recorded | | | | | | None  +  ++  +++ | | | | | 0  1  2  3 |  | | | |  |  |
| 89 | | | 24 hour urinary Output | | | | | | (g/24hr) -------------------------------------- | | | | |  |  | | | |  |  |
| 90 | | | Lowest Urinary Output recorded | | | | | | ------------------------------------------------- | | | | |  |  | | | |  |  |
| 91 | | | Was edema present? | | | | | | Present  Not present | | | | | 1  2 |  | | | |  |  |
| 92 | | | Were convulsions associated (Eclampsia) | | | | | | Yes  No | | | | | 1  2 |  | | | |  |  |
| 93  93.1  93.2  93.4  93.5  93.6 | | | Which other symptoms were present | | | | | | Epigastric and /or liver pair  Headache and / or blurred vision  Pulmonary edema and / or cyanosis  Other specify------------------------------------------------------------------ | | | | | A  B  C  D |  | | | |  |  |
| 94 | | | Was a blood chemistry done( electrolytes and urea) | | | | | | Yes  No | | | | | 1  2 | If no, skip to 96 | | | |  |  |
| 95 | | | If yes, what was the result like? | | | | | | Sodium…………………………..  Potassium……………………….  Chloride…………………………  Bicarbonate………………………  Urea……………………………...  Creatinine………………………..  Others specify………………………………………………………………………………………………………………………………… | | | | |  |  | | | |  |  |
| 96 | | | Was there any indication for termination of pregnancy | | | | | | Yes  No | | | | | 1  2 |  | | | |  |  |
|  | | | **SEVERE HAEMORRHAGE** | | | | | |  | | | | |  |  | | | |  |  |
| 97 | | | Was there Severe Haemorrhage | | | | | | Yes  No | | | | | 1  2 | If no skip to 111 | | | |  |  |
| 98 | | | If yes, what was the primary cause | | | | | |  | | | | |  |  | | | |  |  |
| 99 | | | Estimated blood loss (mls) | | | | | | ………………………. | | | | |  |  | | | |  |  |
| 100 | | | What was the method of estimation? | | | | | | Visual only  Weighted swabs | | | | | 1  2 |  | | | |  |  |
| 101 | | | PCV pre delivery (Nearest to delivery or intrapartum) | | | | | |  | | | | |  |  | | | |  |  |
| 102 | | | PCV Post delivery (first one taken post delivery | | | | | |  | | | | |  |  | | | |  |  |
| 103 | | | Was their associated shock? | | | | | | Yes  No | | | | | 1  2 | If no, skip to 105 | | | |  |  |
| 104 | | | If yes what were the features | | | | | | …………………………………  …………………………………  ………………………………… | | | | |  |  | | | |  |  |
| 105 | | | Was their Emergency hysterectomy | | | | | | Yes  No | | | | | 1  2 | If no skip to 107 | | | |  |  |
| 106 | | | What was the indication for the hysterectomy? | | | | | | Morbidly adherent placenta  Uncontrolled haemorrhage  Uterine rupture  Others specify ……………….  ……………………………….. | | | | | 1  2  3  4 |  | | | |  |  |
| 107 | | | Were their Coagulation defects | | | | | | Yes  No | | | | | 1  2 | If no, skip to 110 | | | |  |  |
| 108 | | | If yes, What was the platelet count? | | | | | | ………………………………. | | | | |  |  | | | |  |  |
| 109 | | | What was the clotting time? | | | | | | ………………………………. | | | | |  |  | | | |  |  |
| 110 | | | Number of units of blood transfused | | | | | | ………………………………… | | | | |  |  | | | |  |  |
|  | | | **DYSTOCIA** | | | | | |  | | | | |  |  | | | |  |  |
| 111 | | | Was there prolonged obstructed labour? | | | | | | Yes  No | | | | | 1  2 | If no, skip to 114 | | | |  |  |
| 112 | | | What was the duration of labour? | | | | | |  | | | | |  |  | | | |  |  |
| 113 | | | Which of these did the woman also Experience? | | | | | | Imminent Uterine Rupture in a previous scar *(i.e lower abdominal pain, thinned out lower uterine segment on surgery*  Ruptured Uterus  Fetal death  Septicaemia  Septic Shock  Others……………………………………………………………….. | | | | | A  B  C  D  E  F |  | | | |  |  |
|  | | | **SEVERE SEPSIS** | | | | | |  | | | | |  |  | | | |  |  |
| 114 | | | Was there septicaemia | | | | | | Yes  No | | | | | 1  2 | If no, skip to 123 | | | |  |  |
| 115 | | | If yes, What caused it type? | | | | | | Peripartum infections  Puerperal sepsis  Others specify…………………..  …………………………………. | | | | | 1  2  3 |  | | | |  |  |
| 116 | | | Where was the primary site of infection | | | | | | …………………………………. | | | | |  |  | | | |  |  |
| 117 | | | What was the highest temperature recorded? | | | | | | .......................................... | | | | |  |  | | | |  |  |
| 118 | | | Was blood culture done | | | | | | Yes  No | | | | | 1  2 | If no, skip to 120 | | | |  |  |
| 119 | | | If yes, which organism was isolated | | | | | | …………………………………... | | | | |  |  | | | |  |  |
| 120 | | | Was endocervical / high vagina swab taken for m/c/s | | | | | | Yes  No | | | | | 1  2 | If no, skip to 122 | | | |  |  |
| 121 | | | If yes, which organisms were isolated | | | | | | …………………………………... | | | | |  |  | | | |  |  |
| 122 | | | White blood cell count | | | | | |  | | | | |  |  | | | |  |  |
|  | | |  | | | | | |  | | | | |  |  | | | |  |  |
| **SEVERE ANAEMIA** | | | | | | | | | | | | | | | | | | |  |  |
| 123 | | | Was there severe aneamia | | | | | Yes  No | | | | | | 1  2 | | If no, skip 128 | | |  |  |
| 124 | | | What was the packed cell volume | | | | |  | | | | | |  | |  | | |  |  |
| 125 | | | What was the primary cause | | | | | …………………………………………………………………………………………………………………………………………. | | | | | |  | |  | | |  |  |
| 126 | | | What were the associated features | | | | | ……………………………………………………………………………………………………………………………………………………………………………. | | | | | |  | |  | | |  |  |
| 127 | | | How many pints of blood were transfused? | | | | | …………………………………  ………………………………… | | | | | |  | |  | | |  |  |
|  | | | **MANAGEMENT OF SEVERE MORBID EVENT** | | | | |  | | | | | |  | |  | | |  |  |
| 128 | | | How did the pregnancy end! | | | | | Spontaneous labour  Indication of labour  C/S following failed induction  Emergency C/S  Instrumental delivery  C/S following failed instrumental delivery | | | | | | A  B  C  D  E  F | |  | | |  |  |
| 129 | | | Was Hysterectomy performed | | | | | Yes  No | | | | | | 1  2 | |  | | |  |  |
| 130 | | | What was the indication for hysterectomy? | | | | | Uterine rupture  Irremovable Placenta  Unstoppable Bleeding  Others specify | | | | | | A  B  C  D | |  | | |  |  |
| 131 | | | Which other procedures were performed? | | | | | ---------------------------------------------------------------------------------- | | | | | |  | |  | | |  |  |
| 132 | | | What was the indication for this procedure/s? | | | | | ------------------------------------------------------------------------------------------------------------------------------------------------------------------------------------------------------------------------------------------------------ | | | | | |  | |  | | |  |  |
| 133  133.1  133.2  133.3  133.4  133.5  133.6  133.7 | | | What were the medications given and name | | | | | Anticonvulsants……………………………………  Anti coagulants ……………………………………  Antibiotics…………………………………………  Antihypertensive…………………………………..  Corticosteroids…………………………………….  Tocolytics …………………………………………  Others specify………………………………………  ……………………………………………………… | | | | | | | | | | |  |  |
| **PATIENT FLOW ANALYSIS FOR NEAR MISS CASES** | | | | | | | | | | | | | | | | | | |  |  |
| 134 | | | What time did you reach the hospital? | | | | | | Date  Time | | | | |  | | |  | |  |  |
| 135 | | | What time did you enter the maternity ward? | | | | | | Date  Time | | | | |  | | |  | |  |  |
| 136 | | | What time did the initial assessment begin | | | | | | Date  Time | | | | |  | | |  | |  |  |
| 137 | | | What time were you first seen by a senior health professional ( i.e a senior registrar/Consultant with sufficient training to diagnose and treat rather than just record signs and symptoms) | | | | | | Date  Time | | | | |  | | |  | |  |  |
| 138 | | | What time did the senior health professional give a verbal or written orders for treatment | | | | | | Date  Time | | | | |  | |  | | |  |  |
| 139 | | | What time was the Near miss condition diagnosed | | | | | | Date  Time | | | | |  | |  | | |  |  |
| 140 | | | What time were medications given, specifically, antibiotics, oxytocin, anticonvulsants etc | | | | | | Date  Time | | | | |  | |  | | |  |  |
| 141 | | | What time was the procedure conducted, specifically, C-section, hysterectomy, laparotomy etc | | | | | | Date  Time | | | | |  | |  | | |  |  |
| 142 | | | What was the time of birth | | | | | | Date  Time | | | | |  | |  | | |  |  |
| 143 | | | What time was the woman discharged (home/ against medical advice/ deceased) | | | | | | Date  Time | | | | |  | |  | | |  |  |
| 144 | | | Was there a delay in making the initial assessment | | | | | | Yes  No | | | | | 1  2 | | If no, skip to 146 | | |  |  |
| 145 | | | If yes, what were the reasons | | | | | | ……………………………………………………………………………………………………… | | | | |  | |  | | |  |  |
| 146 | | | Was there a delay in making a definitive diagnosis | | | | | | Yes  No | | | | | 1  2 | | If no, skip to 148 | | |  |  |
| 147 | | | If yes, what were the reasons | | | | | | Arrived at night  No diagnoses  Incorrect diagnosis  No Ultrasound  Misinterpretation of presenting symptoms.  Others specify…………………………………………………………… | | | | | 1  2  3  4  5  6  7 | |  | | |  |  |
| 148 | | | Was there any delay in commencing treatment after the definitive diagnosis had been made | | | | | | Yes  No | | | | | 1  2 | | If no, skip to 150 | | |  |  |
| 149 | | | If Yes, why? | | | | | | Unavailability of the paediatrician  Unavailability of the anaesthetist  Unavailability of blood  Electricity failure  Unreadiness of the operating theatre  Others specify…………………………………………………………… | | | | | 1  2  3  4  5  6  7 | |  | | |  |  |
|  | | | **COMPLICATIONS OF NEAR MISS MORBIDITY** | | | | | |  | | | | |  | |  | | |  |  |
| 150 | | | Was/were there any severe complication/s following event? | | | | | | Yes  No | | | | | 1  2 | | If no, skip to 154 | | |  |  |
| 151 | | | Cardiovascular:  Respiratory  Renal:  Neurological (other than CVA)  Gynecological: | | | | | | Cerebro-vascular accident  Heart failure (A,H)  Cardiac arrest  Disseminated Intravascular Coagulopathy  Adult Respiratory Distress Syndrome  Pulmonary oedema  Acute Renal Failure  Failure  Cortical blindness (incl. transient)  Infertility following hysterectomy  Others:…………………………………………………………………………………………………………………………………………………………………… | | | | | A  B  C  D  E  F  G  H  I  J  K | |  | | |  |  |
| 152 | | | What was the outcome at discharge: | | | | | | Alive and apparently well  Alive but with some deficit (either physical or psychological)  Persistent vegetative state  Died | | | | | 1  2  3  4 | | |  | |  |  |
| 153 | | | If the patient died, Summarize the events surrounding this outcome | | | | | | | | | | | | | | | |  |  |
| 154 | | | **SUMMARIZE THE NEAR MISS EVENT** | | | | | | | | | | | | | | | |  |  |

**APPENDIX 11**

INDEPTH INTERVIEW QUESTIONNAIRE

1. Give a verbatim account of your last experience with this last pregnancy and delivery.
2. Did you envisage any difficulties with the pregnancy or delivery? If you did, what were the warning signs?
3. In what ways did you plan to handle any difficulties with pregnancy and delivery when they occur?
4. Do you know what have caused the difficulties you encountered with this pregnancy/delivery?
5. Were there ways you could have prevented or avoided any of these difficulties?
6. What roles did your husband play during this experience? How supportive or unsupportive was he?
7. Who were the other relatives that played significant roles during this period and what were these roles?
8. Were there difficulties with sourcing for the funds required for your treatment? How did you get round this problem? Who provided the funds used?
9. Were there problems with accessing care from this health facility?
10. In what ways did this health facility help in solving the difficulties encountered?
11. In what ways did this facility contribute to some of the difficulties encountered?
12. What do you like about the care you received in this facility?
13. What don’t you like about the care you received in this facility?
14. Would you want to obtain care from this facility another time? Yes/No, why?
15. Can you recommend this facility to a friend? Yes/no, why?
